# Comparing balloon-expandable and self-expanding transfemoral transcatheter aortic valve replacement based on subgroups in Germany 2019/2020

**DOI:** 10.1007/s00392-023-02326-w

**Published:** 2023-11-20

**Authors:** Vera Oettinger, Ingo Hilgendorf, Dennis Wolf, Jonathan Rilinger, Alexander Maier, Manfred Zehender, Dirk Westermann, Klaus Kaier, Constantin von zur Mühlen

**Affiliations:** 1 Department of Cardiology and Angiology, University Heart Center, Medical Center–University of Freiburg, Faculty of Medicine, University of Freiburg, Hugstetter Str. 55, 79106 Freiburg, Germany; 2 Center for Big Data Analysis in Cardiology (CeBAC), Department of Cardiology and Angiology, University Heart Center, Medical Center–University of Freiburg, Faculty of Medicine, University of Freiburg, Freiburg, Germany; 3 Institute of Medical Biometry and Statistics, Faculty of Medicine and Medical Center–University of Freiburg, Freiburg, Germany

**Keywords:** Aortic valve stenosis, Transcatheter aortic valve replacement, Transcatheter aortic valve implantation, In-hospital mortality, Subgroup analysis, National electronic health records

## Abstract

**Background:**

Previously, overall comparable outcomes were seen for balloon-expandable (BE) or self-expanding (SE) transfemoral transcatheter aortic valve replacement (TAVR). However, subgroup analyses based on large case numbers are still needed.

**Methods:**

German national data of all BE and SE transfemoral TAVR treating aortic valve stenosis in 2019 and 2020 were analysed. We then compared different outcomes and performed a subgroup analysis for the endpoint in-hospital mortality.

**Results:**

Overall, 46,243 TAVR were analysed, 19,910 BE, and 26,333 SE. Patients in the SE group had a significantly higher logistic EuroSCORE (13.61 vs 12.66%, *p* < 0.001), age (81.55 vs 79.99a, *p* < 0.001), and proportion of women (54.82 vs 40.06%, *p* < 0.001). Both groups showed a similar in-hospital mortality with 2.37% in BE and 2.35% in SE (*p* = 0.916). In-hospital mortality also did not differ significantly after risk adjustment (OR = 0.98 [0.86, 1.13], *p* = 0.799). Patients in the SE group had a significantly lower risk of major bleeding (OR = 0.83 [0.73, 0.95], *p* = 0.006), but a significantly higher risk of stroke (OR = 1.38 [1.19, 1.59], *p* < 0.001), delirium (OR = 1.15 [1.06, 1.24], *p* = 0.001), and permanent pacemaker implantation (OR = 1.29 [1.21, 1.37], *p* < 0.001). In the subgroup analysis of in-hospital mortality, there were no significant differences in any of the observed subgroups (age < 75/75–79/80–84/ ≥ 85a, logistic EuroSCORE < 4/4– < 9/ ≥ 9, gender, NYHA III/IV, previous CABG, peripheral vascular disease, COPD, pulmonary hypertension, renal disease GFR < 30 ml/min, and diabetes mellitus).

**Conclusion:**

In the direct comparison of balloon-expandable and self-expanding TAVR, there are no differences for in-hospital mortality in subgroups.

**Graphical abstract:**

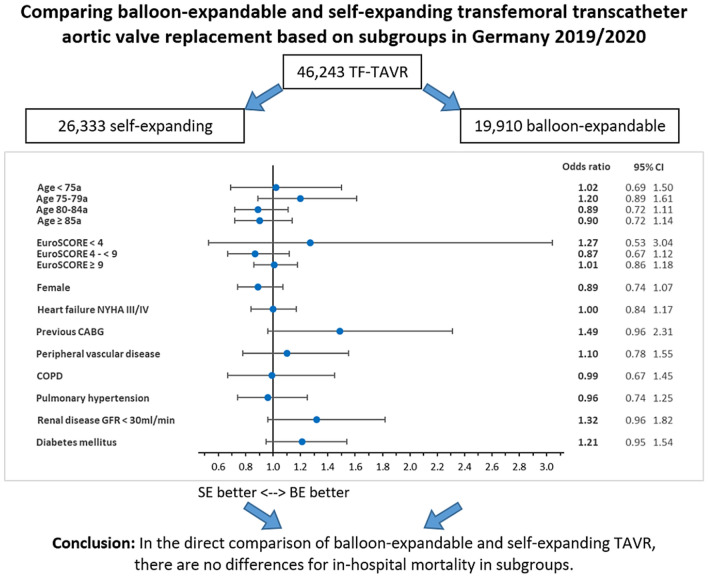

**Supplementary Information:**

The online version contains supplementary material available at 10.1007/s00392-023-02326-w.

## Introduction

The question remains whether there is a difference in outcomes between balloon-expandable (BE) and self-expanding (SE) devices in transcatheter aortic valve replacement (TAVR). In a previous analysis [1], we found overall comparable results for BE as well as SE transfemoral (TF-)TAVR in Germany. In a randomized study, Thiele et al. [2] also demonstrated concordant outcomes in a subgroup analysis for the primary endpoint of all-cause mortality, stroke, moderate and severe paravalvular leakage, as well as permanent pacemaker implantation within 30 days. On the other hand, a French analysis by Deharo et al. [3] showed better results for BE TAVR, which corresponds to Van Belle et al. [4], who also saw this in a subgroup analysis for a composite endpoint of at least moderate paravalvular regurgitation and in-hospital mortality. The international CENTER study observed a lower rate of conversion to open heart surgery, stroke as well as pacemaker implantation and a higher bleeding rate in new-generation BE TAVR, but no difference in in-hospital mortality or within 30 days [5]. Subsequently, the authors also saw no difference between subgroups for mortality and stroke within one year except for valve sizes > 26 mm with lower mortality in BE TAVR [6]. In addition, regarding valve-in-valve procedures, they found lower bleeding rates within 30 days in SE TAVR, but again no difference in mortality [7].

Taking these studies together, outcomes of BE and SE TAVR appear to be overall comparable. In order to investigate this subsequently, further subgroup analyses based on large case numbers are needed. Therefore, we now compared all balloon-expandable and self-expanding transfemoral TAVR for aortic stenosis in Germany in 2019 or 2020, and formed subgroups for the endpoint in-hospital mortality.

## Materials and methods

Since 2005, the German Federal Statistical Office, by means of its Research Data Centre DESTATIS, has provided the data on all patient stays in German hospitals. Those are based on inpatient hospital charges within the German DRG system. The DRG system makes use of fixed charge groups, which are formed based on diagnoses (coded according to ICD-10) respectively procedures performed (coded according to OPS).

Data on 46,243 cases of TAVR procedures performed in 2019 or 2020 were extracted from this database. As described in a previous study, patients with a baseline diagnosis of pure aortic regurgitation (principal or secondary diagnosis other than I35.0, I35.2, I06.0, I06.2) were excluded [1, 8].

In our analysis, the investigators did not have a direct access to data from individual patients. They solely had access to summary results provided by the Research Data Centre. Therefore, according to German law, ethics committee approval as well as informed consent were not required. All pooled results have been anonymized by DESTATIS. This means that any information that could be used to identify an individual patient or a single center was censored by DESTATIS to ensure privacy. In addition, the data are checked and censored by DESTATIS to prevent any conclusions being drawn about an individual center.

The analysis focused on nine differing endpoints: in-hospital mortality, major bleeding events, stroke, acute kidney injury, postoperative delirium, mechanical ventilation > 48 h, permanent pacemaker implantation, length of hospital stay, and reimbursement. Stroke as well as acute kidney injury were defined through ICD, Tenth Revision (ICD-10) codes (secondary diagnosis I63* or I64 and N17*).

Major bleeding was defined as the need for transfusion of > 5 units of red blood cells (OPS codes 8–800.c1 to 8–800.cr). In-hospital mortality, duration of mechanical ventilation, length of hospital stay, and reimbursement were part of the main DESTATIS variable set. Regarding all other comorbidities, the existing anamnestic or acute specific codes were used (OPS and ICD codes have been discussed in detail previously [1, 8]).

For the calculation of the estimated logistic EuroSCORE (European System for Cardiac Operative Risk Evaluation), all fields could be filled in except for critical preoperative status and left ventricular function. For these, we presumed a normal status (i.e., no critical preoperative status and no left ventricular dysfunction), thus calculated a best-case scenario.

*p* values were calculated on the basis of Student's *t* tests or chi-square tests. In previous studies [1, 8], a number of baseline characteristics were identified for description of the risk profiles between the treatment groups. As there was no randomization of patients to the two observed procedural options (use of balloon-expandable or self-expanding TAVR), we applied multivariable logistic or linear regression models with use of these baseline characteristics included as potential confounders (see Table 1 for the covariates listed). Furthermore, a random intercept at center level was used to account for the correlation of error terms for patients that were treated in the same center. The results of the different regression analyses are shown in Supplementary Appendix 1. Missing values could not be imputed in this analysis as there were no codes to indicate any missing data. If the electronic health record of a patient did not contain information on a specific clinical characteristic, it was presumed to be absent. No adjustment was made for multiple testing. Hence, *p* values should not be interpreted as confirmatory, but are of a descriptive nature. Furthermore, inferences that are drawn from the 95% confidence intervals may not be reproducible.

**Table 1:** Baseline characteristics of patients treated with balloon-expandable or self-expanding transfemoral TAVR in Germany in 2019 and 2020

	Balloon-expandable TAVR		Self-expanding TAVR		*p* value
*N*	19,910		26,333		
Conducted in 2020 (instead of 2019)	52.40%		49.90%		< 0.001
Logistic EuroSCORE, mean / SD	12.66	9.88	13.61	10.10	< 0.001
Age in years, mean / SD	79.99	6.54	81.55	5.73	< 0.001
Female	40.06%		54.82%		< 0.001
NYHA II	14.70%		13.78%		0.005
NYHA III or IV	50.33%		50.43%		0.824
CAD	55.43%		52.57%		< 0.001
Arterial hypertension	62.52%		63.41%		0.048
Previous MI within 4 months	1.62%		1.30%		0.004
Previous MI within 1 year	0.70%		0.55%		0.038
Previous MI after 1 year	4.74%		3.59%		< 0.001
Previous CABG	7.58%		7.20%		0.117
Previous cardiac surgery	13.51%		12.83%		0.031
Peripheral vascular disease	10.22%		8.33%		< 0.001
Carotid disease	6.91%		5.40%		< 0.001
COPD	10.66%		10.29%		0.202
Pulmonary hypertension	21.04%		19.54%		< 0.001
Renal disease, GFR < 15 ml/min	2.60%		2.00%		< 0.001
Renal disease, GFR < 30 ml/min	3.89%		4.07%		0.318
Atrial fibrillation	44.15%		44.44%		0.541
Diabetes mellitus	30.79%		31.29%		0.256
Emergency	9.97%		10.32%		0.215
Number of cases per center, mean / SD	379.37	178.97	382.43	174.09	0.065

*CABG* coronary artery bypass graft; *CAD* coronary artery disease; *COPD* chronic obstructive pulmonary disease; *EuroSCORE* European system for cardiac operative risk evaluation; *GFR* glomerular filtration rate; *MI* myocardial infarction; *N* number of procedures; *NYHA* New York Heart Association; *SD* standard deviation

Following previous approaches on the topic of subgroup analyses in aortic valve replacement [9, 10], predefined subgroups were examined for subgroup-specific treatment effects: age < 75/75–79/80–84/ ≥ 85a, logistic EuroSCORE < 4/4– < 9/ ≥ 9, gender, NYHA III/IV (New York Heart Association), previous coronary artery bypass graft (CABG), peripheral vascular disease, chronic obstructive pulmonary disease (COPD), pulmonary hypertension, renal disease with glomerular filtration rate (GFR) < 30 ml/min, and diabetes mellitus.

All analyses were performed using Stata 17 (StataCorp, College Station, Texas, USA).

## Results

### Baseline characteristics

Overall, 46,243 transfemoral TAVR procedures for aortic valve stenosis were performed in 2019 or 2020, with 19,910 balloon-expandable and 26,333 self-expanding valves (Table 1). Patients in the SE group had a significantly higher logistic EuroSCORE of 13.61 vs 12.66% in BE (*p* < 0.001), age of 81.55 vs 79.99a (*p* < 0.001), and proportion of women of 54.82 vs 40.06% (*p* < 0.001). Furthermore, there were significantly more patients in the BE group with coronary artery disease with 55.43 vs 52.57% in SE (*p* < 0.001), peripheral vascular disease with 10.22 vs 8.33% (*p* < 0.001), as well as carotid disease with 6.91 vs 5.40% (*p* < 0.001).

### Unadjusted in-hospital outcomes of balloon-expandable or self-expanding TAVR in 2019/2020

Regarding the unadjusted in-hospital mortality (Table 2), both groups showed a similar mortality rate of 2.37% in BE and 2.35% in SE (*p* = 0.916). Patients treated with BE TF-TAVR had a significantly higher rate of major bleeding (2.81 vs 2.31%, *p* = 0.001). However, the BE group showed a lower rate of stroke (1.85 vs 2.55%, *p* < 0.001), delirium (7.22 vs 8.74%, *p* < 0.001), and permanent pacemaker implantation (12.13 vs 14.62%, *p* < 0.001). No difference was seen in acute kidney injury (9.49 vs 9.56%, *p* = 0.798) and mechanical ventilation > 48 h (2.24 vs 2.10%, *p* = 0.305).

**Table 2:** Unadjusted in-hospital outcomes of balloon-expandable or self-expanding TAVR in 2019/2020

	Balloon-expandable TAVR		Self-expanding TAVR		*p* value BE vs SE
*N*	19,910		26,333		
In-hospital mortality	2.37%		2.35%		0.916
Major bleeding > 5 units	2.81%		2.31%		0.001
Stroke	1.85%		2.55%		< 0.001
Acute kidney injury	9.49%		9.56%		0.798
Delirium	7.22%		8.74%		< 0.001
Mechanical ventilation > 48 h	2.24%		2.10%		0.305
Permanent pacemaker implantation	12.13%		14.62%		< 0.001
Length of hospital stay (mean, SD)	11.86d	8.37d	11.61d	8.04d	0.001
Reimbursement (mean, SD)	26,223€	6,226€	26,131€	5,991€	0.108

*BE* balloon-expandable; *N* number of procedures; *SD* standard deviation; *SE* self-expanding

Regarding resource utilization parameters, patients receiving SE TF-TAVR had a significantly shorter length of hospital stay (11.86 vs 11.61 days, *p* = 0.001). There was no difference in reimbursement (26,223 vs 26,131 €, *p* = 0.108).

### Risk-adjusted in-hospital outcomes of balloon-expandable or self-expanding TAVR in 2019/2020

**Fig. 1:**
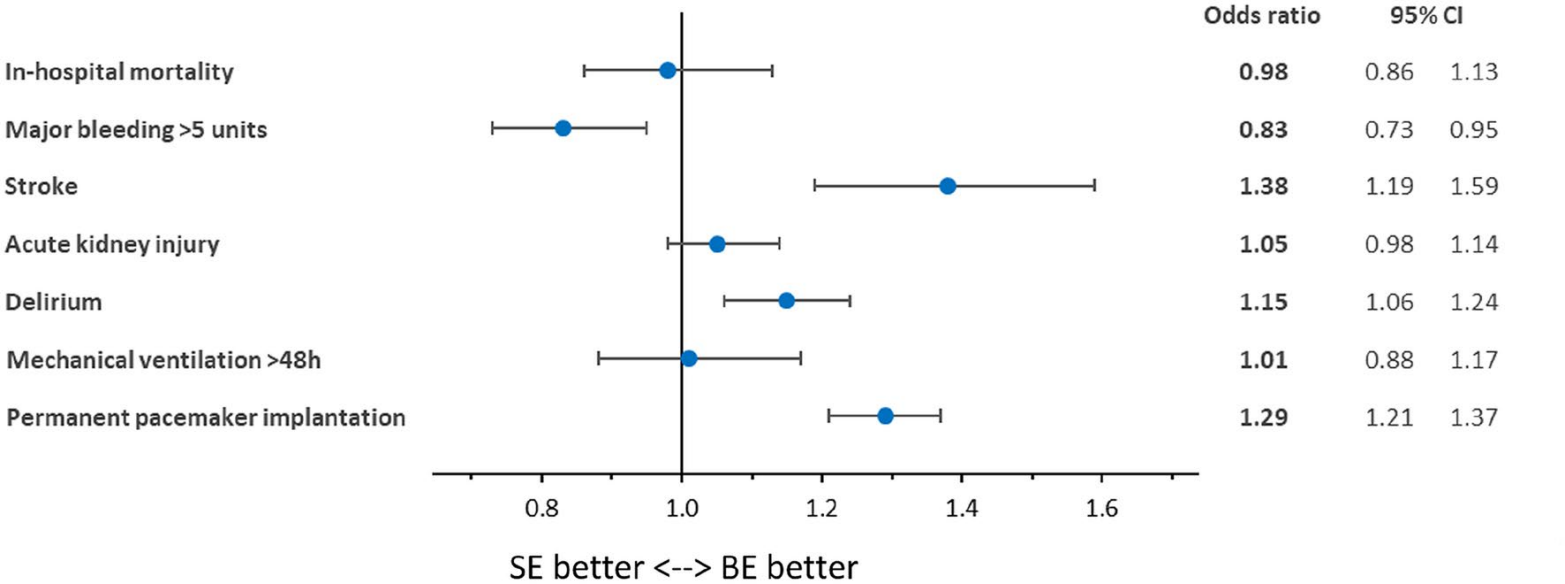
Risk-adjusted in-hospital outcomes of self-expanding instead of balloon-expandable TAVR in 2019 and 2020. *BE* balloon-expandable; *CI* confidence interval; *SE* self-expanding

In-hospital mortality also did not differ significantly after risk adjustment (risk-adjusted OR = 0.98 [95% CI 0.86, 1.13], *p* = 0.799; Fig. 1). Patients receiving SE TF-TAVR had a significantly lower risk of major bleeding (OR = 0.83 [0.73, 0.95], *p* = 0.006). However, they had a significantly higher risk of stroke (OR = 1.38 [1.19, 1.59], *p* < 0.001), delirium (OR = 1.15 [1.06, 1.24], *p* = 0.001), and permanent pacemaker implantation (OR = 1.29 [1.21, 1.37], *p* < 0.001). There was no significant difference after risk adjustment for acute kidney injury (OR = 1.05 [0.98, 1.14], *p* = 0.180) and mechanical ventilation > 48 h (OR = 1.01 [0.88, 1.17], *p* = 0.871). Resource utilization parameters also did not differ regarding length of hospital stay (risk-adjusted Coefficient = 0.19d [− 0.01d, 0.39d], *p* = 0.066) and reimbursement (Coefficient = 117€ [− 22€, 257€], *p* = 0.099; Table 3).

**Table 3:** Risk-adjusted in-hospital outcomes as well as resource utilization parameters of self-expanding instead of balloon-expandable TAVR in 2019/2020

	OR	*p* value	95% CI	
In-hospital mortality	0.98	0.799	0.86	1.13
Major bleeding > 5 units	0.83	0.006	0.73	0.95
Stroke	1.38	< 0.001	1.19	1.59
Acute kidney injury	1.05	0.180	0.98	1.14
Delirium	1.15	0.001	1.06	1.24
Mechanical ventilation > 48 h	1.01	0.871	0.88	1.17
Permanent pacemaker implantation	1.29	< 0.001	1.21	1.37

	Coefficient	*p* value	95% CI	
Length of hospital stay	0.19d	0.066	-0.01d	0.39d
Reimbursement	117€	0.099	-22€	257€

*CI* confidence interval; *OR* odds ratio

Primary risk factors regarding in-hospital mortality were higher grade renal disease (GFR < 15 ml/min: OR = 2.67 [2.04, 3.50], *p* < 0.001; GFR < 30 ml/min: OR = 1.59 [1.24, 2.04], *p* < 0.001), higher grade heart failure NYHA III/IV (OR = 1.81 [1.56, 2.10], *p* < 0.001), and atrial fibrillation (OR = 1.43 [1.26, 1.62], *p* < 0.001; Supplementary Appendix 1).

### Risk-adjusted subgroup analysis for the endpoint in-hospital mortality comparing balloon-expandable and self-expanding TAVR

**Fig. 2:**
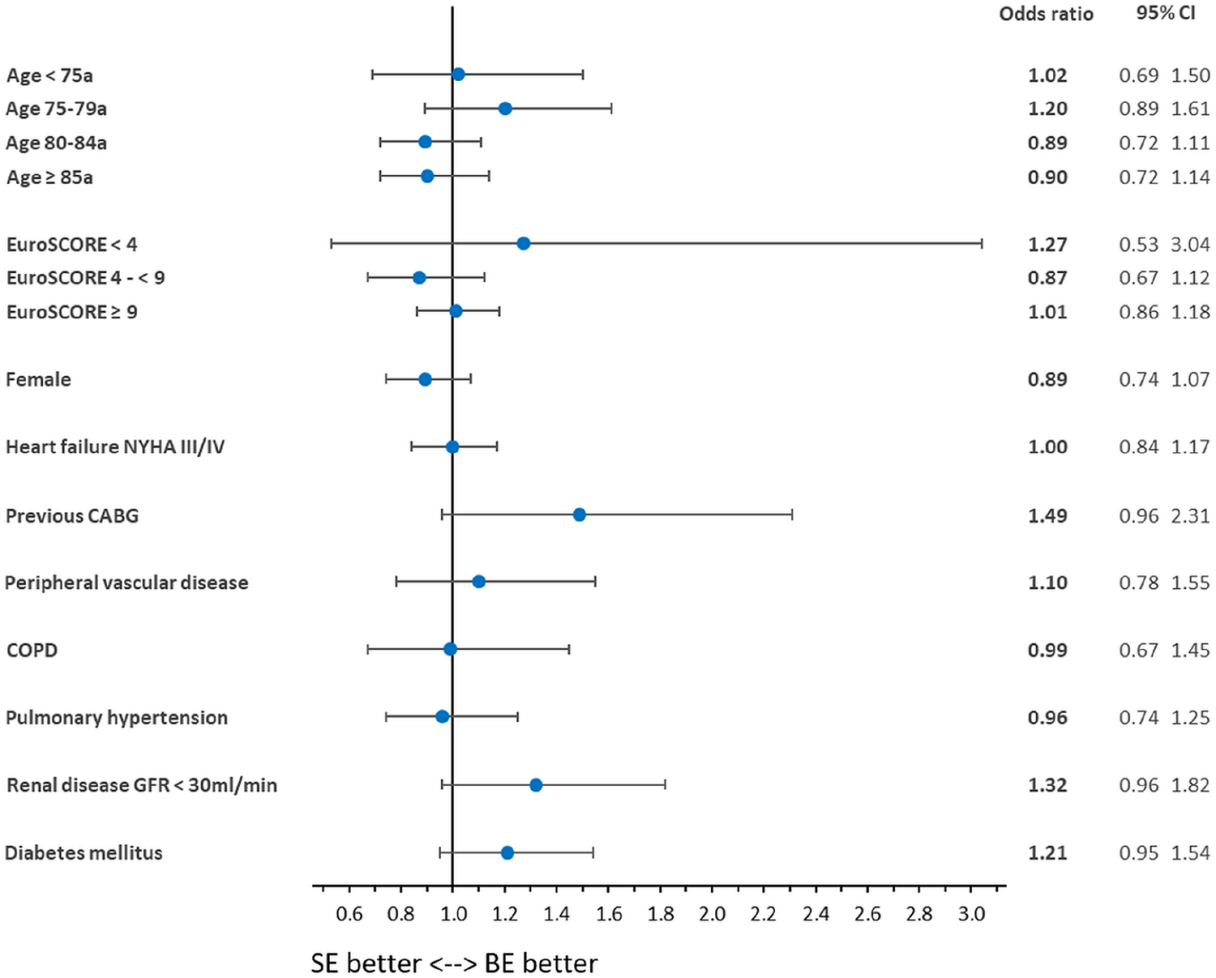
Risk-adjusted subgroup analysis for the endpoint in-hospital mortality of self-expanding instead of balloon-expandable TAVR in 2019 and 2020. *BE* balloon-expandable; *CI* confidence interval; *SE* self-expanding

Furthermore, we performed a subgroup analysis for the endpoint in-hospital mortality. The full regression analysis can be found in the Supplementary Appendix 2. Looking at the endpoint in-hospital mortality (Table 4, Fig. 2), there were no significant differences between SE and BE TF-TAVR in any of the subgroups: age < 75a (SE instead of BE: OR = 1.02 [0.69, 1.50], *p* = 0.935), 75-79a (OR = 1.20 [0.89, 1.61], *p* = 0.225), 80-84a (OR = 0.89 [0.72, 1.11], *p* = 0.294), ≥ 85a (OR = 0.90 [0.72, 1.14], *p* = 0.386); EuroSCORE < 4 (OR = 1.27 [0.53, 3.04], *p* = 0.594), 4- < 9 (OR = 0.87 [0.67, 1.12], *p* = 0.280), ≥ 9 (OR = 1.01 [0.86, 1.18], *p* = 0.941); gender (OR = 0.89 [0.74, 1.07], *p* = 0.204); NYHA III/IV (OR = 1.00 [0.84, 1.17], *p* = 0.957); previous CABG (OR = 1.49 [0.96, 2.31], *p* = 0.073); peripheral vascular disease (OR = 1.10 [0.78, 1.55], *p* = 0.588); COPD (OR = 0.99 [0.67, 1.45], *p* = 0.957); pulmonary hypertension (OR = 0.96 [0.74, 1.25], *p* = 0.779); renal disease GFR < 30 ml/min (OR = 1.32 [0.96, 1.82], *p* = 0.091); diabetes mellitus (OR = 1.21 [0.95, 1.54], *p* = 0.128).

**Table 4:** Subgroup analysis for the endpoint in-hospital mortality: Self-expanding instead of balloon-expandable TAVR in 2019/2020

	*N*	% SE	OR	*p *value	95% CI	
Age < 75a	5,625	43.40%	1.02	0.935	0.69	1.50
Age 75-79a	10,489	53.57%	1.20	0.225	0.89	1.61
Age 80-84a	18,028	59.31%	0.89	0.294	0.72	1.11
Age ≥ 85a	12,101	62.64%	0.90	0.386	0.72	1.14
EuroSCORE < 4	2.198	38.44%	1.27	0.594	0.53	3.04
EuroSCORE 4- < 9	17,854	55.66%	0.87	0.280	0.67	1.12
EuroSCORE ≥ 9	26,191	59.38%	1.01	0.941	0.86	1.18
Female	22,412	64.42%	0.89	0.204	0.74	1.07
NYHA III/IV	23,300	57.00%	1.00	0.957	0.84	1.17
Previous CABG	3,406	55.67%	1.49	0.073	0.96	2.31
Peripheral vascular disease	4,228	51.89%	1.10	0.588	0.78	1.55
COPD	4,832	56.08%	0.99	0.957	0.67	1.45
Pulmonary hypertension	9,335	55.12%	0.96	0.779	0.74	1.25
Renal disease GFR < 30 ml/min	2,890	55.30%	1.32	0.091	0.96	1.82
Diabetes mellitus	14,370	57.33%	1.21	0.128	0.95	1.54

*CABG* coronary artery bypass graft; *CI* confidence interval; *COPD* chronic obstructive pulmonary disease; *EuroSCORE* European System for Cardiac Operative Risk Evaluation; *GFR* glomerular filtration rate; *N* number of procedures; *NYHA* New York Heart Association; *OR* odds ratio; *SD* standard deviation; *SE* self-expanding

## Discussion

In our analysis of 46,243 BE and SE TF-TAVR in Germany in 2019 or 2020, we found that patients who received SE TF-TAVR had a significantly lower risk of major bleeding, but showed a significantly higher risk of stroke, delirium, and permanent pacemaker implantation. However, there were no differences for in-hospital mortality in the subgroup analysis.

In a previous study [1], we saw overall comparable results for BE as well as SE TF-TAVR in Germany in 2018. Risk of permanent pacemaker implantation differed significantly towards better results in BE TAVR. This corresponds to our current analysis. ORs for major bleeding, stroke, and delirium were not significant in the previous study. However, there were similar trends of lower bleeding rates and higher rates of stroke and delirium in SE TAVR, which were significant in our current analysis. This can be explained by the fact that we now analysed an even larger number of cases over two years instead of just one year. Length of hospital stay was significantly shorter for SE TAVR in both analyses. However, it should be noted that the overall differences are rather small. Furthermore, both analyses showed similar results for in-hospital mortality.

Consistent with our findings, the CENTER study also found lower rates of stroke and permanent pacemaker implantation as well as a higher rate of major or life-threatening bleeding in new-generation BE TAVR. However, there was no significant difference in mortality during the hospital admission or within 30 days [5], which fits our results. In addition, another analysis of the CENTER study investigated valve-in-valve TAVR and showed lower rates of major bleeding within 30 days with SE TAVR. However, the authors again saw no difference in mortality [7]. Furthermore, Habertheuer et al. [11] observed higher stroke rates within 30 days in SE TAVR, but no difference in midterm stroke rates. They also found no difference in mortality, readmission, renal failure, pacemaker implantation as well as paravalvular regurgitation within 30 days, and no difference in midterm mortality as well as readmission. In addition, Abdel-Wahab et al. [12] showed comparable clinical results for mortality, stroke, repeat hospitalization due to heart failure, myocardial infarction, bleeding events, and vascular complications for BE and SE TAVR in the German CHOICE-study in a 5-year follow-up, with lower pacemaker implantation rates in BE TAVR, but better forward-flow hemodynamics in SE valves.

Our data showed a mean permanent pacemaker implantation rate of 12.13% in BE and 14.62% in SE TAVR. Reviews in the literature have also analysed this topic. Bruno et al. [13] found values from 6.7 to 39.2% and the pooled incidence was 19%. van Rosendael et al. [14] saw mean rates between 2.3% and 36.1% for new-generation devices within a peri-procedural to 2-year follow-up, 4.0–24.0% for new-generation BE prostheses and 14.7–26.7% for SE. In addition, analyses of the German Aortic Valve Registry (GARY) revealed rates of 12.6% in BE and 19.5% in SE current-generation TAVR devices within the hospital stay [15] and 13.0–21.9% in second-generation valves at 1 year [16].

Regarding the further complications, a GARY analysis [15] revealed a lower in-hospital rate of disabling stroke of 1.1% in BE and 1.2% in SE TAVR. However, we analysed every coded stroke, not just disabling ones. In addition, the authors observed a blood transfusion rate of > 4 units of 1.4% in both BE and SE TAVR. We found rates of 2.81% in BE and 2.31% in SE TAVR requiring > 5 units. It should be noted that the data provided by DESTATIS cannot be used to distinguish whether an in-hospital complication was directly related to TAVR or whether it was a complication related to another procedure during the same hospital stay.

In our subgroup analysis comparing BE and SE TAVR for the endpoint in-hospital mortality, we did not find significant differences in any of the observed subgroups. Our data therefore suggest that, regarding the endpoint in-hospital mortality, the choice between BE or SE valves in case of a TF-TAVR can be made almost independently of the observed characteristics or pre-existing conditions of patients. The European [17] and American [18] guidelines for the management of valvular heart disease also do not specify whether BE or SE valves might be the better choice in the presence of certain patient characteristics or pre-existing conditions [7]. Other factors, such as anatomical conditions of the individual patient, may be relevant to valve selection [1, 2], as also mentioned in the American guideline [18].

Our German analysis contrasts with the French results of Van Belle et al. [4], who found better outcomes with BE TAVR in a subgroup analysis for a composite endpoint of at least moderate paravalvular regurgitation and in-hospital mortality. However, our results are consistent with the German SOLVE-TAVI trial by Thiele et al. [2], which found similar outcomes for BE and SE TAVR in a subgroup analysis for the primary endpoint consisting of all-cause mortality, stroke, moderate and severe paravalvular leakage as well as permanent pacemaker implantation within 30 days. In addition, the CENTER study also found no significant differences between the observed subgroups for mortality and stroke within one year except for valve sizes > 26 mm with a lower mortality rate in BE TAVR. The authors therefore conclude that mortality and stroke rates within one year were similar for BE as well as SE TAVR [6].

Strengths and limitations of this study are in accordance with previous analyses [1, 19–23]. The strength is the evaluation of complete German national data comparing all balloon-expandable and self-expanding transfemoral TAVR for aortic valve stenosis. Limitation of administrative data is possible coding error. The analysed factors depend on the coded values and for example reimbursement may influence the coding. In addition, we can only approximate Valve Academic Research Consortium 3 (VARC-3) criteria [24]. Because of the characteristics of the data, a long-term follow-up, e.g. outcomes within 1 year, cannot be performed. Finally, we cannot assure the inclusion of all relevant parameters in our study and some information—e.g. the precise valve type, anatomical conditions, pacemaker indications, causes of in-hospital mortality or a differentiation of BE and SE procedures of the same center—is not available in the data set.

## Conclusions

In conclusion, we compared outcomes of 46,243 balloon-expandable and self-expanding TAVR for aortic valve stenosis in Germany in 2019 or 2020, and performed a subgroup analysis for the endpoint in-hospital mortality. Patients receiving SE TF-TAVR had a lower risk of major bleeding, but a higher risk of stroke, delirium, and permanent pacemaker implantation. However, there were no differences for in-hospital mortality in all observed subgroups.

### Supplementary Information

Below is the link to the electronic supplementary material.

Supplementary file1 (PDF 644 KB)

## Data Availability

Data are available upon reasonable request. The patients’ data are stored on the server of the Federal Bureau of statistics and not available due to data protection. The calculated raw data are sent anonymized to the scientist.
