# Supplementary material for: Comparing balloon-expandable and self-expanding transfemoral transcatheter aortic valve replacement based on subgroups in Germany 2019/2020
Source: Clin Res Cardiol. 2023 Nov 20;113(1):168–76. doi: 10.1007/s00392-023-02326-w (PMC10808194; doi:10.1007/s00392-023-02326-w)
Supplement: Supplementary file 1 — Supplementary file1 (PDF 644 KB) [file 392_2023_2326_MOESM1_ESM.pdf]

## Comparing balloon-expandable and self-expanding transfemoral transcatheter aortic valve replacement based on subgroups in Germany 2019/2020

Vera Oettinger, MD, M.Sc.; Ingo Hilgendorf, MD; Dennis Wolf, MD; Jonathan Rilinger, MD; Alexander Maier, MD; Manfred Zehender, MD, PhD; Dirk Westermann, MD; Klaus Kaier, PhD; Constantin von zur Mühlen, MD

**Supplementary Appendix 1:** Regression results of patients treated with balloon-expandable or self-expanding transfemoral TAVR in Germany in 2019 and 2020

All patients (N=46,243)

| | In-hospital mortality | | | | Bleeding (>5 units) | | | | Stroke | | | |
|---|---|---|---|---|---|---|---|---|---|---|---|---|
| | OR | p-value | 95%CI | | OR | p-value | 95%CI | | OR | p-value | 95%CI | |
| **Self-expanding (instead of balloon-expandable)** | **0.98** | **0.799** | **0.86** | **1.13** | **0.83** | **0.006** | **0.73** | **0.95** | **1.38** | **0.000** | **1.19** | **1.59** |
| **Conducted in 2020 (instead of 2019)** | 0.94 | 0.302 | 0.83 | 1.06 | 0.93 | 0.228 | 0.83 | 1.05 | 1.10 | 0.128 | 0.97 | 1.25 |
| **Logistic EuroSCORE** | 1.03 | 0.000 | 1.02 | 1.05 | 1.01 | 0.117 | 1.00 | 1.02 | 1.17 | 0.000 | 1.16 | 1.18 |
| **Age in years** | 1.00 | 0.683 | 0.98 | 1.01 | 0.97 | 0.000 | 0.96 | 0.98 | 0.91 | 0.000 | 0.90 | 0.92 |
| **Female** | 1.05 | 0.468 | 0.92 | 1.21 | 1.33 | 0.000 | 1.17 | 1.52 | 0.69 | 0.000 | 0.60 | 0.79 |
| **NYHA II** | 0.35 | 0.000 | 0.25 | 0.49 | 0.62 | 0.000 | 0.49 | 0.80 | 0.93 | 0.497 | 0.75 | 1.15 |
| **NYHA III or IV** | 1.81 | 0.000 | 1.56 | 2.10 | 1.64 | 0.000 | 1.42 | 1.90 | 1.12 | 0.149 | 0.96 | 1.29 |
| **CAD** | 1.12 | 0.102 | 0.98 | 1.27 | 1.20 | 0.005 | 1.06 | 1.37 | 1.07 | 0.329 | 0.93 | 1.23 |
| **Hypertension** | 0.71 | 0.000 | 0.62 | 0.82 | 0.71 | 0.000 | 0.62 | 0.82 | 1.02 | 0.816 | 0.88 | 1.18 |
| **Previous MI within 4 months** | 0.52 | 0.030 | 0.29 | 0.94 | 1.07 | 0.785 | 0.67 | 1.69 | 0.39 | 0.000 | 0.24 | 0.63 |
| **Previous MI within 1 year** | 0.91 | 0.806 | 0.45 | 1.87 | 1.01 | 0.964 | 0.53 | 1.95 | 0.84 | 0.687 | 0.36 | 1.94 |
| **Previous MI after 1 year** | 1.08 | 0.580 | 0.82 | 1.44 | 0.75 | 0.078 | 0.55 | 1.03 | 0.80 | 0.195 | 0.56 | 1.12 |
| **Previous CABG** | 0.65 | 0.005 | 0.49 | 0.88 | 0.46 | 0.000 | 0.34 | 0.62 | 0.79 | 0.189 | 0.55 | 1.13 |
| **Previous cardiac surgery** | 1.12 | 0.449 | 0.83 | 1.51 | 1.74 | 0.000 | 1.32 | 2.31 | 0.11 | 0.000 | 0.08 | 0.15 |
| **Peripheral vascular disease** | 1.24 | 0.048 | 1.00 | 1.53 | 1.47 | 0.000 | 1.20 | 1.81 | 0.38 | 0.000 | 0.30 | 0.48 |
| **Carotid disease** | 0.89 | 0.375 | 0.69 | 1.15 | 1.39 | 0.005 | 1.10 | 1.75 | 0.50 | 0.000 | 0.39 | 0.64 |
| **COPD** | 0.85 | 0.133 | 0.69 | 1.05 | 0.93 | 0.483 | 0.76 | 1.14 | 0.36 | 0.000 | 0.29 | 0.45 |
| **Pulmonary hypertension** | 0.75 | 0.006 | 0.61 | 0.92 | 0.88 | 0.234 | 0.72 | 1.08 | 0.13 | 0.000 | 0.10 | 0.16 |
| **Renal disease, GFR <15mg/dl** | 2.67 | 0.000 | 2.04 | 3.50 | 3.14 | 0.000 | 2.43 | 4.05 | 0.29 | 0.000 | 0.19 | 0.44 |
| **Renal disease, GFR <30mg/dl** | 1.59 | 0.000 | 1.24 | 2.04 | 2.03 | 0.000 | 1.58 | 2.61 | 0.13 | 0.000 | 0.09 | 0.18 |
| **Atrial fibrillation** | 1.43 | 0.000 | 1.26 | 1.62 | 1.62 | 0.000 | 1.43 | 1.83 | 1.21 | 0.004 | 1.06 | 1.38 |
| **Diabetes mellitus** | 0.92 | 0.219 | 0.80 | 1.05 | 0.86 | 0.020 | 0.75 | 0.98 | 1.11 | 0.120 | 0.97 | 1.28 |
| **Emergency** | 1.19 | 0.099 | 0.97 | 1.47 | 1.45 | 0.000 | 1.18 | 1.78 | 0.25 | 0.000 | 0.20 | 0.32 |
| **Number of cases per center** | 1.00 | 0.000 | 1.00 | 1.00 | 1.00 | 0.012 | 1.00 | 1.00 | 1.00 | 0.383 | 1.00 | 1.00 |

| | Acute kidney injury | | | | Postoperative delirium | | | | Mechanical ventilation >48h | | | |
|---|---|---|---|---|---|---|---|---|---|---|---|---|
| | OR | p-value | 95%CI | | OR | p-value | 95%CI | | OR | p-value | 95%CI | |
| **Self-expanding (instead of balloon-expandable)** | **1.05** | **0.180** | **0.98** | **1.14** | **1.15** | **0.001** | **1.06** | **1.24** | **1.01** | **0.871** | **0.88** | **1.17** |
| **Conducted in 2020 (instead of 2019)** | 0.97 | 0.424 | 0.91 | 1.04 | 0.93 | 0.055 | 0.87 | 1.00 | 0.83 | 0.004 | 0.73 | 0.94 |
| **Logistic EuroSCORE** | 1.02 | 0.000 | 1.01 | 1.03 | 1.02 | 0.000 | 1.01 | 1.03 | 1.03 | 0.000 | 1.02 | 1.04 |
| **Age in years** | 1.00 | 0.235 | 0.99 | 1.00 | 1.03 | 0.000 | 1.03 | 1.04 | 0.94 | 0.000 | 0.93 | 0.96 |
| **Female** | 0.88 | 0.001 | 0.81 | 0.95 | 0.64 | 0.000 | 0.59 | 0.69 | 0.94 | 0.377 | 0.81 | 1.08 |
| **NYHA II** | 0.90 | 0.087 | 0.79 | 1.02 | 0.91 | 0.138 | 0.81 | 1.03 | 0.50 | 0.000 | 0.36 | 0.68 |
| **NYHA III or IV** | 2.06 | 0.000 | 1.90 | 2.24 | 1.23 | 0.000 | 1.13 | 1.34 | 1.92 | 0.000 | 1.64 | 2.24 |
| **CAD** | 1.18 | 0.000 | 1.10 | 1.27 | 1.09 | 0.023 | 1.01 | 1.17 | 1.27 | 0.001 | 1.11 | 1.46 |
| **Hypertension** | 0.88 | 0.002 | 0.82 | 0.95 | 1.01 | 0.886 | 0.93 | 1.09 | 0.76 | 0.000 | 0.65 | 0.87 |
| **Previous MI within 4 months** | 1.10 | 0.465 | 0.86 | 1.40 | 0.68 | 0.016 | 0.50 | 0.93 | 0.75 | 0.299 | 0.44 | 1.29 |
| **Previous MI within 1 year** | 0.91 | 0.651 | 0.62 | 1.35 | 0.72 | 0.167 | 0.45 | 1.15 | 0.74 | 0.453 | 0.34 | 1.62 |
| **Previous MI after 1 year** | 1.05 | 0.533 | 0.90 | 1.22 | 0.90 | 0.253 | 0.76 | 1.07 | 0.86 | 0.362 | 0.63 | 1.18 |
| **Previous CABG** | 0.81 | 0.024 | 0.68 | 0.97 | 0.67 | 0.000 | 0.55 | 0.83 | 0.52 | 0.000 | 0.38 | 0.72 |
| **Previous cardiac surgery** | 0.97 | 0.748 | 0.81 | 1.16 | 0.85 | 0.107 | 0.69 | 1.04 | 1.10 | 0.540 | 0.81 | 1.48 |
| **Peripheral vascular disease** | 1.11 | 0.091 | 0.98 | 1.26 | 0.94 | 0.357 | 0.82 | 1.08 | 1.23 | 0.066 | 0.99 | 1.52 |
| **Carotid disease** | 1.14 | 0.058 | 1.00 | 1.31 | 1.05 | 0.568 | 0.90 | 1.22 | 0.90 | 0.474 | 0.69 | 1.19 |
| **COPD** | 1.06 | 0.322 | 0.95 | 1.18 | 0.85 | 0.011 | 0.75 | 0.96 | 1.23 | 0.037 | 1.01 | 1.48 |
| **Pulmonary hypertension** | 1.07 | 0.266 | 0.95 | 1.19 | 0.80 | 0.000 | 0.70 | 0.90 | 0.76 | 0.010 | 0.61 | 0.94 |
| **Renal disease, GFR <15mg/dl** | 0.92 | 0.432 | 0.74 | 1.14 | 1.45 | 0.001 | 1.17 | 1.79 | 2.00 | 0.000 | 1.51 | 2.65 |
| **Renal disease, GFR <30mg/dl** | 3.84 | 0.000 | 3.35 | 4.39 | 0.86 | 0.103 | 0.71 | 1.03 | 1.37 | 0.022 | 1.05 | 1.80 |
| **Atrial fibrillation** | 1.59 | 0.000 | 1.48 | 1.70 | 1.49 | 0.000 | 1.39 | 1.59 | 2.18 | 0.000 | 1.90 | 2.50 |
| **Diabetes mellitus** | 1.53 | 0.000 | 1.42 | 1.63 | 1.18 | 0.000 | 1.09 | 1.27 | 1.12 | 0.094 | 0.98 | 1.28 |
| **Emergency** | 1.61 | 0.000 | 1.43 | 1.81 | 1.11 | 0.141 | 0.97 | 1.27 | 1.26 | 0.032 | 1.02 | 1.57 |
| **Number of cases per center** | 1.00 | 0.611 | 1.00 | 1.00 | 1.00 | 0.097 | 1.00 | 1.00 | 1.00 | 0.000 | 1.00 | 1.00 |

| | Permanent pacemaker implantation | | | | Length of stay | | | | Reimbursement | | | |
|---|---|---|---|---|---|---|---|---|---|---|---|---|
| | OR | p-value | 95%CI | | Coeff | p-value | 95%CI | | Coeff | p-value | 95%CI | |
| **Self-expanding (instead of balloon-expandable)** | **1.29** | **0.000** | **1.21** | **1.37** | **0.19** | **0.066** | **-0.01** | **0.39** | **117** | **0.099** | **-22** | **257** |
| **Conducted in 2020 (instead of 2019)** | 1.01 | 0.793 | 0.95 | 1.06 | -0.82 | 0.000 | -1.05 | -0.59 | -2,326 | 0.000 | -2,471 | -2,180 |
| **Logistic EuroSCORE** | 1.00 | 0.751 | 0.99 | 1.01 | 0.12 | 0.000 | 0.09 | 0.14 | 44 | 0.000 | 22 | 67 |
| **Age in years** | 1.02 | 0.000 | 1.01 | 1.02 | -0.08 | 0.000 | -0.10 | -0.06 | -72.84 | 0.000 | -92.36 | -53.31 |
| **Female** | 0.74 | 0.000 | 0.70 | 0.79 | 0.20 | 0.012 | 0.04 | 0.35 | -235 | 0.000 | -343 | -127 |
| **NYHA II** | 0.95 | 0.235 | 0.87 | 1.04 | 0.15 | 0.320 | -0.14 | 0.44 | 68 | 0.398 | -90 | 227 |
| **NYHA III or IV** | 1.04 | 0.253 | 0.97 | 1.11 | 1.76 | 0.000 | 1.34 | 2.18 | 707 | 0.000 | 486 | 928 |
| **CAD** | 0.99 | 0.754 | 0.93 | 1.05 | 0.44 | 0.000 | 0.27 | 0.62 | 181 | 0.000 | 87 | 276 |
| **Hypertension** | 0.98 | 0.599 | 0.92 | 1.05 | -0.28 | 0.030 | -0.53 | -0.03 | -311 | 0.003 | -517 | -104 |
| **Previous MI within 4 months** | 1.04 | 0.730 | 0.83 | 1.30 | -0.92 | 0.007 | -1.59 | -0.25 | -522 | 0.022 | -968 | -76 |
| **Previous MI within 1 year** | 1.01 | 0.950 | 0.72 | 1.42 | 0.17 | 0.695 | -0.70 | 1.05 | -301 | 0.395 | -995 | 393 |
| **Previous MI after 1 year** | 1.04 | 0.546 | 0.91 | 1.19 | 0.02 | 0.909 | -0.36 | 0.41 | -89 | 0.579 | -404 | 226 |
| **Previous CABG** | 0.59 | 0.000 | 0.51 | 0.68 | -2.11 | 0.000 | -2.85 | -1.37 | -757 | 0.000 | -1,164 | -351 |
| **Previous cardiac surgery** | 1.54 | 0.000 | 1.33 | 1.79 | 0.16 | 0.670 | -0.58 | 0.90 | 76 | 0.684 | -289 | 440 |
| **Peripheral vascular disease** | 1.01 | 0.844 | 0.91 | 1.13 | 0.42 | 0.076 | -0.04 | 0.87 | 241 | 0.150 | -87 | 568 |
| **Carotid disease** | 1.02 | 0.749 | 0.90 | 1.16 | 0.04 | 0.813 | -0.32 | 0.41 | -93 | 0.479 | -351 | 165 |
| **COPD** | 0.97 | 0.537 | 0.88 | 1.07 | 0.22 | 0.163 | -0.09 | 0.53 | 72 | 0.609 | -205 | 349 |
| **Pulmonary hypertension** | 0.98 | 0.699 | 0.89 | 1.08 | -0.51 | 0.000 | -0.79 | -0.23 | -338 | 0.016 | -613 | -64 |
| **Renal disease, GFR <15mg/dl** | 1.26 | 0.012 | 1.05 | 1.50 | 3.47 | 0.000 | 2.36 | 4.58 | 2,104 | 0.000 | 988 | 3,219 |
| **Renal disease, GFR <30mg/dl** | 1.14 | 0.074 | 0.99 | 1.33 | 2.67 | 0.000 | 1.92 | 3.43 | 942 | 0.009 | 233 | 1,650 |
| **Atrial fibrillation** | 1.14 | 0.000 | 1.08 | 1.21 | 1.64 | 0.000 | 1.44 | 1.84 | 681 | 0.000 | 545 | 817 |
| **Diabetes mellitus** | 1.14 | 0.000 | 1.08 | 1.21 | 0.63 | 0.000 | 0.44 | 0.83 | 182 | 0.007 | 49 | 315 |
| **Emergency** | 1.13 | 0.032 | 1.01 | 1.26 | 4.40 | 0.000 | 3.52 | 5.27 | 1,022 | 0.000 | 587 | 1,457 |
| **Number of cases per center** | 1.00 | 0.210 | 1.00 | 1.00 | -0.01 | 0.004 | -0.01 | 0.00 | -3 | 0.076 | -6 | 0 |

CABG: coronary artery bypass graft; CAD: coronary artery disease; COPD: chronic obstructive pulmonary disease; EuroSCORE: European System for Cardiac Operative Risk Evaluation; GFR: glomerular filtration rate; MI: myocardial infarction; NYHA: New York Heart Association; SAVR: surgical aortic valve replacement; SD: standard deviation; TAVR: transcatheter aortic valve replacement.

**Supplementary Appendix 2:** Risk-adjusted subgroup analysis for the endpoint in-hospital mortality of patients treated with balloon-expandable or self-expanding transfemoral TAVR in Germany in 2019 and 2020

| | In-hospital mortality | | | | In-hospital mortality | | | | In-hospital mortality | | | | In-hospital mortality | | | |
|---|---|---|---|---|---|---|---|---|---|---|---|---|---|---|---|---|
| **Subgroup** | **Age < 75a** | | | | **Age 75-79a** | | | | **Age 80-84a** | | | | **Age ≥ 85a** | | | |
| **N** | 5,625 | | | | 10,489 | | | | 18,028 | | | | 12,101 | | | |
| **% Self-expanding** | 43.40% | | | | 53.57% | | | | 59.31% | | | | 62.64% | | | |
| | **OR** | **p-value** | **95%CI** | | **OR** | **p-value** | **95%CI** | | **OR** | **p-value** | **95%CI** | | **OR** | **p-value** | **95%CI** | |
| **Self-expanding (instead of balloon-expandable)** | 1.02 | 0.935 | 0.69 | 1.50 | 1.20 | 0.225 | 0.89 | 1.61 | 0.89 | 0.294 | 0.72 | 1.11 | 0.90 | 0.386 | 0.72 | 1.14 |
| **Conducted in 2020 (instead of 2019)** | 1.15 | 0.455 | 0.79 | 1.68 | 0.74 | 0.036 | 0.56 | 0.98 | 1.09 | 0.386 | 0.89 | 1.34 | 0.87 | 0.195 | 0.71 | 1.07 |
| **Logistic EuroSCORE** | 1.03 | 0.188 | 0.99 | 1.07 | 1.04 | 0.015 | 1.01 | 1.07 | 1.04 | 0.001 | 1.02 | 1.06 | 1.05 | 0.000 | 1.03 | 1.07 |
| **Age in years** | 0.96 | 0.058 | 0.93 | 1.00 | 0.96 | 0.393 | 0.86 | 1.06 | 1.06 | 0.147 | 0.98 | 1.14 | 1.00 | 0.898 | 0.96 | 1.05 |
| **Female** | 1.24 | 0.317 | 0.82 | 1.87 | 1.10 | 0.570 | 0.80 | 1.50 | 0.84 | 0.126 | 0.66 | 1.05 | 1.13 | 0.328 | 0.88 | 1.46 |
| **NYHA II** | 0.25 | 0.010 | 0.09 | 0.72 | 0.34 | 0.007 | 0.15 | 0.74 | 0.40 | 0.000 | 0.24 | 0.66 | 0.29 | 0.000 | 0.15 | 0.55 |
| **NYHA III or IV** | 1.27 | 0.266 | 0.83 | 1.95 | 2.08 | 0.000 | 1.47 | 2.94 | 1.61 | 0.000 | 1.26 | 2.05 | 1.75 | 0.000 | 1.35 | 2.26 |
| **CAD** | 1.41 | 0.107 | 0.93 | 2.12 | 1.10 | 0.524 | 0.82 | 1.49 | 1.06 | 0.623 | 0.85 | 1.32 | 1.12 | 0.319 | 0.90 | 1.40 |
| **Hypertension** | 0.94 | 0.762 | 0.64 | 1.39 | 0.67 | 0.008 | 0.50 | 0.90 | 0.77 | 0.022 | 0.62 | 0.96 | 0.70 | 0.002 | 0.56 | 0.88 |
| **Previous MI within 4 months** | 0.40 | 0.373 | 0.05 | 3.01 | 1.00 | xx | xx | xx | 0.92 | 0.839 | 0.40 | 2.09 | 0.38 | 0.066 | 0.14 | 1.07 |
| **Previous MI within 1 year** | 0.84 | 0.870 | 0.11 | 6.47 | 0.43 | 0.412 | 0.06 | 3.24 | 0.95 | 0.936 | 0.30 | 3.07 | 1.54 | 0.476 | 0.47 | 5.03 |
| **Previous MI after 1 year** | 2.00 | 0.033 | 1.06 | 3.78 | 1.35 | 0.328 | 0.74 | 2.49 | 0.62 | 0.104 | 0.35 | 1.10 | 1.11 | 0.671 | 0.68 | 1.82 |
| **Previous CABG** | 0.50 | 0.095 | 0.22 | 1.13 | 0.86 | 0.632 | 0.46 | 1.60 | 0.67 | 0.113 | 0.40 | 1.10 | 0.73 | 0.275 | 0.42 | 1.28 |
| **Previous cardiac surgery** | 1.14 | 0.730 | 0.54 | 2.42 | 1.06 | 0.860 | 0.56 | 2.02 | 0.98 | 0.936 | 0.58 | 1.65 | 0.68 | 0.198 | 0.38 | 1.22 |
| **Peripheral vascular disease** | 2.01 | 0.010 | 1.18 | 3.42 | 0.96 | 0.855 | 0.59 | 1.55 | 1.19 | 0.339 | 0.83 | 1.70 | 0.84 | 0.414 | 0.55 | 1.28 |
| **Carotid disease** | 0.78 | 0.565 | 0.34 | 1.80 | 1.04 | 0.894 | 0.59 | 1.83 | 0.82 | 0.382 | 0.54 | 1.27 | 0.71 | 0.163 | 0.45 | 1.15 |
| **COPD** | 0.93 | 0.779 | 0.55 | 1.56 | 0.99 | 0.971 | 0.64 | 1.53 | 0.74 | 0.126 | 0.51 | 1.09 | 0.66 | 0.047 | 0.44 | 0.99 |
| **Pulmonary hypertension** | 1.05 | 0.872 | 0.60 | 1.83 | 0.69 | 0.118 | 0.43 | 1.10 | 0.70 | 0.053 | 0.49 | 1.00 | 0.54 | 0.002 | 0.36 | 0.80 |
| **Renal disease, GFR <15mg/dl** | 1.70 | 0.089 | 0.92 | 3.14 | 3.35 | 0.000 | 1.94 | 5.77 | 2.62 | 0.000 | 1.57 | 4.36 | 2.43 | 0.002 | 1.38 | 4.30 |
| **Renal disease, GFR <30mg/dl** | 2.48 | 0.013 | 1.21 | 5.09 | 1.76 | 0.060 | 0.98 | 3.16 | 2.05 | 0.001 | 1.36 | 3.08 | 0.80 | 0.335 | 0.50 | 1.27 |
| **Atrial fibrillation** | 1.52 | 0.035 | 1.03 | 2.23 | 1.61 | 0.001 | 1.21 | 2.15 | 1.39 | 0.002 | 1.13 | 1.71 | 1.37 | 0.004 | 1.11 | 1.70 |
| **Diabetes mellitus** | 0.67 | 0.046 | 0.45 | 0.99 | 0.93 | 0.616 | 0.69 | 1.25 | 1.03 | 0.797 | 0.83 | 1.28 | 0.90 | 0.409 | 0.70 | 1.16 |
| **Emergency** | 2.37 | 0.001 | 1.41 | 3.98 | 1.33 | 0.236 | 0.83 | 2.11 | 1.03 | 0.889 | 0.71 | 1.48 | 0.81 | 0.294 | 0.55 | 1.20 |
| **Number of cases per center** | 1.00 | 0.034 | 1.00 | 1.00 | 1.00 | 0.004 | 1.00 | 1.00 | 1.00 | 0.000 | 1.00 | 1.00 | 1.00 | 0.022 | 1.00 | 1.00 |

| | In-hospital mortality | | | | In-hospital mortality | | | | In-hospital mortality | | | | In-hospital mortality | | | |
|---|---|---|---|---|---|---|---|---|---|---|---|---|---|---|---|---|
| **Subgroup** | **EuroSCORE < 4** | | | | **EuroSCORE 4 - < 9** | | | | **EuroSCORE ≥ 9** | | | | **Female** | | | |
| **N** | 2,198 | | | | 17,854 | | | | 26,191 | | | | 22,412 | | | |
| **% Self-expanding** | 38.44% | | | | 55.66% | | | | 59.38% | | | | 64.42% | | | |
| | **OR** | **p-value** | **95%CI** | | **OR** | **p-value** | **95%CI** | | **OR** | **p-value** | **95%CI** | | **OR** | **p-value** | **95%CI** | |
| **Self-expanding (instead of balloon-expandable)** | 1.27 | 0.594 | 0.53 | 3.04 | 0.87 | 0.280 | 0.67 | 1.12 | 1.01 | 0.941 | 0.86 | 1.18 | 0.89 | 0.204 | 0.74 | 1.07 |
| **Conducted in 2020 (instead of 2019)** | 1.64 | 0.269 | 0.68 | 3.95 | 0.98 | 0.876 | 0.76 | 1.26 | 0.91 | 0.221 | 0.79 | 1.06 | 0.89 | 0.177 | 0.75 | 1.05 |
| **Logistic EuroSCORE** | 0.52 | 0.166 | 0.21 | 1.31 | 1.06 | 0.662 | 0.82 | 1.38 | 1.05 | 0.000 | 1.03 | 1.06 | 1.04 | 0.000 | 1.02 | 1.05 |
| **Age in years** | 0.99 | 0.871 | 0.90 | 1.09 | 1.00 | 0.952 | 0.90 | 1.10 | 0.97 | 0.003 | 0.95 | 0.99 | 1.00 | 0.854 | 0.98 | 1.02 |
| **Female** | 1.48 | 0.493 | 0.48 | 4.58 | 1.09 | 0.766 | 0.63 | 1.87 | 0.89 | 0.168 | 0.75 | 1.05 | 1.00 | xx | xx | xx |
| **NYHA II** | 1.00 | xx | xx | xx | 0.30 | 0.000 | 0.16 | 0.58 | 0.38 | 0.000 | 0.25 | 0.56 | 0.34 | 0.000 | 0.21 | 0.55 |
| **NYHA III or IV** | 2.14 | 0.110 | 0.84 | 5.46 | 1.87 | 0.000 | 1.40 | 2.48 | 1.71 | 0.000 | 1.43 | 2.04 | 1.64 | 0.000 | 1.34 | 2.02 |
| **CAD** | 1.56 | 0.317 | 0.65 | 3.73 | 1.10 | 0.468 | 0.85 | 1.43 | 1.10 | 0.213 | 0.94 | 1.29 | 1.19 | 0.058 | 0.99 | 1.42 |
| **Hypertension** | 0.40 | 0.041 | 0.16 | 0.96 | 0.57 | 0.000 | 0.44 | 0.73 | 0.78 | 0.003 | 0.67 | 0.92 | 0.73 | 0.001 | 0.61 | 0.88 |
| **Previous MI within 4 months** | 1.00 | xx | xx | xx | 0.73 | 0.774 | 0.08 | 6.28 | 0.41 | 0.005 | 0.22 | 0.76 | 0.50 | 0.135 | 0.20 | 1.24 |
| **Previous MI within 1 year** | 5.55 | 0.156 | 0.52 | 59.28 | 0.64 | 0.664 | 0.09 | 4.74 | 0.84 | 0.674 | 0.36 | 1.92 | 1.18 | 0.757 | 0.42 | 3.27 |
| **Previous MI after 1 year** | 1.00 | xx | xx | xx | 1.04 | 0.910 | 0.52 | 2.07 | 1.13 | 0.442 | 0.83 | 1.54 | 0.96 | 0.873 | 0.59 | 1.57 |
| **Previous CABG** | 1.00 | xx | xx | xx | 1.00 | xx | xx | xx | 0.64 | 0.004 | 0.47 | 0.86 | 0.73 | 0.210 | 0.45 | 1.19 |
| **Previous cardiac surgery** | 16.89 | 0.081 | 0.70 | 406.05 | 1.00 | xx | xx | xx | 0.78 | 0.138 | 0.56 | 1.08 | 1.04 | 0.868 | 0.68 | 1.58 |
| **Peripheral vascular disease** | 20.89 | 0.003 | 2.73 | 159.86 | 1.64 | 0.399 | 0.52 | 5.13 | 0.97 | 0.809 | 0.77 | 1.23 | 1.02 | 0.887 | 0.74 | 1.42 |
| **Carotid disease** | 1.00 | xx | xx | xx | 0.44 | 0.312 | 0.09 | 2.15 | 0.74 | 0.032 | 0.56 | 0.97 | 0.81 | 0.294 | 0.55 | 1.19 |
| **COPD** | 4.33 | 0.060 | 0.94 | 20.03 | 0.76 | 0.559 | 0.30 | 1.92 | 0.69 | 0.002 | 0.54 | 0.87 | 0.80 | 0.170 | 0.59 | 1.10 |
| **Pulmonary hypertension** | 1.00 | xx | xx | xx | 0.59 | 0.466 | 0.15 | 2.42 | 0.57 | 0.000 | 0.44 | 0.72 | 0.72 | 0.029 | 0.53 | 0.97 |
| **Renal disease, GFR <15mg/dl** | 1.00 | xx | xx | xx | 1.62 | 0.481 | 0.42 | 6.19 | 2.23 | 0.000 | 1.65 | 3.01 | 2.39 | 0.000 | 1.53 | 3.72 |
| **Renal disease, GFR <30mg/dl** | 1.00 | xx | xx | xx | 4.02 | 0.023 | 1.21 | 13.36 | 1.20 | 0.185 | 0.92 | 1.58 | 1.59 | 0.008 | 1.13 | 2.24 |
| **Atrial fibrillation** | 0.59 | 0.367 | 0.19 | 1.85 | 1.68 | 0.000 | 1.31 | 2.17 | 1.35 | 0.000 | 1.17 | 1.57 | 1.38 | 0.000 | 1.16 | 1.64 |
| **Diabetes mellitus** | 0.33 | 0.052 | 0.11 | 1.01 | 0.92 | 0.558 | 0.70 | 1.21 | 0.94 | 0.440 | 0.80 | 1.10 | 0.77 | 0.010 | 0.63 | 0.94 |
| **Emergency** | 1.00 | xx | xx | xx | 1.57 | 0.475 | 0.45 | 5.45 | 0.91 | 0.434 | 0.71 | 1.16 | 0.97 | 0.848 | 0.71 | 1.32 |
| **Number of cases per center** | 1.00 | 0.069 | 0.99 | 1.00 | 1.00 | 0.000 | 1.00 | 1.00 | 1.00 | 0.001 | 1.00 | 1.00 | 1.00 | 0.000 | 1.00 | 1.00 |

| | In-hospital mortality | | | | In-hospital mortality | | | | In-hospital mortality | | | | In-hospital mortality | | | |
|---|---|---|---|---|---|---|---|---|---|---|---|---|---|---|---|---|
| **Subgroup** | **NYHA III/IV** | | | | **Previous CABG** | | | | **Peripheral vascular disease** | | | | **COPD** | | | |
| **N** | 23,300 | | | | 3,406 | | | | 4,228 | | | | 4,832 | | | |
| **% Self-expanding** | 57.00% | | | | 55.67% | | | | 51.89% | | | | 56.08% | | | |
| | **OR** | **p-value** | **95%CI** | | **OR** | **p-value** | **95%CI** | | **OR** | **p-value** | **95%CI** | | **OR** | **p-value** | **95%CI** | |
| **Self-expanding (instead of balloon-expandable)** | 1.00 | 0.957 | 0.84 | 1.17 | 1.49 | 0.073 | 0.96 | 2.31 | 1.10 | 0.588 | 0.78 | 1.55 | 0.99 | 0.957 | 0.67 | 1.45 |
| **Conducted in 2020 (instead of 2019)** | 1.01 | 0.905 | 0.87 | 1.17 | 0.83 | 0.367 | 0.55 | 1.25 | 0.91 | 0.566 | 0.65 | 1.26 | 0.78 | 0.167 | 0.54 | 1.11 |
| **Logistic EuroSCORE** | 1.03 | 0.000 | 1.02 | 1.05 | 1.04 | 0.070 | 1.00 | 1.08 | 1.05 | 0.001 | 1.02 | 1.08 | 1.02 | 0.279 | 0.99 | 1.05 |
| **Age in years** | 0.99 | 0.376 | 0.98 | 1.01 | 0.99 | 0.642 | 0.93 | 1.04 | 0.96 | 0.030 | 0.92 | 1.00 | 0.99 | 0.535 | 0.95 | 1.03 |
| **Female** | 1.00 | 0.958 | 0.85 | 1.19 | 0.98 | 0.933 | 0.57 | 1.67 | 0.72 | 0.088 | 0.49 | 1.05 | 1.02 | 0.932 | 0.68 | 1.53 |
| **NYHA II** | 0.60 | 0.165 | 0.30 | 1.23 | 0.33 | 0.037 | 0.11 | 0.94 | 0.37 | 0.040 | 0.14 | 0.95 | 0.39 | 0.054 | 0.15 | 1.02 |
| **NYHA III or IV** | 1.00 | xx | xx | xx | 1.36 | 0.211 | 0.84 | 2.21 | 2.22 | 0.000 | 1.44 | 3.43 | 1.24 | 0.323 | 0.81 | 1.92 |
| **CAD** | 1.14 | 0.100 | 0.97 | 1.34 | 0.55 | 0.061 | 0.29 | 1.03 | 0.94 | 0.723 | 0.65 | 1.35 | 1.09 | 0.672 | 0.74 | 1.59 |
| **Hypertension** | 0.86 | 0.072 | 0.73 | 1.01 | 0.90 | 0.624 | 0.58 | 1.38 | 1.24 | 0.251 | 0.86 | 1.79 | 0.85 | 0.418 | 0.58 | 1.26 |
| **Previous MI within 4 months** | 0.59 | 0.113 | 0.30 | 1.13 | 1.00 | xx | xx | xx | 0.71 | 0.529 | 0.25 | 2.04 | 1.00 | xx | xx | xx |
| **Previous MI within 1 year** | 0.84 | 0.679 | 0.37 | 1.93 | 0.80 | 0.831 | 0.10 | 6.27 | 1.00 | xx | xx | xx | 0.87 | 0.897 | 0.11 | 6.80 |
| **Previous MI after 1 year** | 1.00 | 0.978 | 0.72 | 1.41 | 1.51 | 0.144 | 0.87 | 2.62 | 1.08 | 0.801 | 0.59 | 1.98 | 1.57 | 0.183 | 0.81 | 3.04 |
| **Previous CABG** | 0.65 | 0.017 | 0.46 | 0.93 | 1.00 | xx | xx | xx | 0.79 | 0.528 | 0.38 | 1.65 | 0.60 | 0.282 | 0.24 | 1.51 |
| **Previous cardiac surgery** | 1.01 | 0.955 | 0.71 | 1.44 | 1.00 | xx | xx | xx | 0.44 | 0.055 | 0.19 | 1.02 | 0.89 | 0.793 | 0.37 | 2.14 |
| **Peripheral vascular disease** | 1.38 | 0.010 | 1.08 | 1.76 | 0.85 | 0.647 | 0.43 | 1.69 | 1.00 | xx | xx | xx | 1.35 | 0.304 | 0.76 | 2.38 |
| **Carotid disease** | 0.94 | 0.711 | 0.70 | 1.28 | 0.49 | 0.115 | 0.20 | 1.19 | 1.32 | 0.226 | 0.84 | 2.05 | 1.32 | 0.395 | 0.70 | 2.48 |
| **COPD** | 0.78 | 0.048 | 0.61 | 1.00 | 0.51 | 0.105 | 0.23 | 1.15 | 0.60 | 0.058 | 0.35 | 1.02 | 1.00 | xx | xx | xx |
| **Pulmonary hypertension** | 0.73 | 0.010 | 0.57 | 0.93 | 0.52 | 0.113 | 0.23 | 1.17 | 0.55 | 0.042 | 0.31 | 0.98 | 1.21 | 0.523 | 0.68 | 2.17 |
| **Renal disease, GFR <15mg/dl** | 2.46 | 0.000 | 1.78 | 3.40 | 0.83 | 0.759 | 0.26 | 2.70 | 3.08 | 0.000 | 1.69 | 5.61 | 2.77 | 0.012 | 1.25 | 6.12 |
| **Renal disease, GFR <30mg/dl** | 1.48 | 0.008 | 1.11 | 1.98 | 1.95 | 0.112 | 0.86 | 4.45 | 1.37 | 0.322 | 0.73 | 2.58 | 1.68 | 0.159 | 0.82 | 3.47 |
| **Atrial fibrillation** | 1.47 | 0.000 | 1.26 | 1.71 | 1.64 | 0.025 | 1.06 | 2.54 | 1.15 | 0.420 | 0.82 | 1.60 | 1.55 | 0.022 | 1.07 | 2.25 |
| **Diabetes mellitus** | 0.87 | 0.078 | 0.74 | 1.02 | 1.25 | 0.294 | 0.82 | 1.91 | 1.02 | 0.911 | 0.73 | 1.43 | 1.09 | 0.640 | 0.75 | 1.58 |
| **Emergency** | 1.09 | 0.519 | 0.85 | 1.39 | 1.36 | 0.406 | 0.66 | 2.83 | 1.14 | 0.640 | 0.65 | 2.00 | 1.74 | 0.075 | 0.95 | 3.21 |
| **Number of cases per center** | 1.00 | 0.000 | 1.00 | 1.00 | 1.00 | 0.180 | 1.00 | 1.00 | 1.00 | 0.435 | 1.00 | 1.00 | 1.00 | 0.042 | 1.00 | 1.00 |

| | In-hospital mortality | | | | In-hospital mortality | | | | In-hospital mortality | | | |
|---|---|---|---|---|---|---|---|---|---|---|---|---|
| **Subgroup** | **Pulmonary hypertension** | | | | **Renal disease GFR <30ml/min** | | | | **Diabetes mellitus** | | | |
| **N** | 9,335 | | | | 2,890 | | | | 14,370 | | | |
| **% Self-expanding** | 55.12% | | | | 55.30% | | | | 57.33% | | | |
| | **OR** | **p-value** | **95%CI** | | **OR** | **p-value** | **95%CI** | | **OR** | **p-value** | **95%CI** | |
| **Self-expanding (instead of balloon-expandable)** | 0.96 | 0.779 | 0.74 | 1.25 | 1.32 | 0.091 | 0.96 | 1.82 | 1.21 | 0.128 | 0.95 | 1.54 |
| **Conducted in 2020 (instead of 2019)** | 0.79 | 0.064 | 0.62 | 1.01 | 0.96 | 0.784 | 0.71 | 1.30 | 0.86 | 0.175 | 0.69 | 1.07 |
| **Logistic EuroSCORE** | 1.03 | 0.009 | 1.01 | 1.05 | 1.01 | 0.372 | 0.98 | 1.04 | 1.04 | 0.000 | 1.02 | 1.06 |
| **Age in years** | 0.99 | 0.444 | 0.96 | 1.02 | 1.00 | 0.931 | 0.96 | 1.03 | 1.00 | 0.798 | 0.97 | 1.02 |
| **Female** | 0.99 | 0.956 | 0.74 | 1.33 | 0.96 | 0.823 | 0.68 | 1.37 | 0.78 | 0.045 | 0.61 | 0.99 |
| **NYHA II** | 0.66 | 0.141 | 0.38 | 1.15 | 0.26 | 0.005 | 0.10 | 0.67 | 0.39 | 0.002 | 0.22 | 0.70 |
| **NYHA III or IV** | 1.67 | 0.002 | 1.21 | 2.31 | 1.16 | 0.417 | 0.81 | 1.68 | 1.65 | 0.000 | 1.26 | 2.16 |
| **CAD** | 0.97 | 0.850 | 0.75 | 1.27 | 1.03 | 0.852 | 0.74 | 1.44 | 1.21 | 0.128 | 0.95 | 1.55 |
| **Hypertension** | 1.10 | 0.501 | 0.83 | 1.45 | 0.88 | 0.434 | 0.64 | 1.21 | 0.97 | 0.815 | 0.76 | 1.25 |
| **Previous MI within 4 months** | 0.67 | 0.452 | 0.23 | 1.92 | 0.72 | 0.619 | 0.20 | 2.59 | 0.58 | 0.224 | 0.24 | 1.40 |
| **Previous MI within 1 year** | 1.00 | xx | xx | xx | 0.32 | 0.262 | 0.04 | 2.36 | 0.46 | 0.290 | 0.11 | 1.93 |
| **Previous MI after 1 year** | 1.81 | 0.011 | 1.15 | 2.87 | 0.81 | 0.511 | 0.43 | 1.53 | 1.14 | 0.566 | 0.73 | 1.76 |
| **Previous CABG** | 0.64 | 0.130 | 0.35 | 1.14 | 0.76 | 0.435 | 0.38 | 1.52 | 0.80 | 0.401 | 0.49 | 1.34 |
| **Previous cardiac surgery** | 0.86 | 0.639 | 0.46 | 1.60 | 1.19 | 0.659 | 0.55 | 2.56 | 0.89 | 0.679 | 0.52 | 1.53 |
| **Peripheral vascular disease** | 1.24 | 0.310 | 0.82 | 1.90 | 1.71 | 0.024 | 1.07 | 2.73 | 1.31 | 0.118 | 0.93 | 1.84 |
| **Carotid disease** | 0.84 | 0.495 | 0.51 | 1.39 | 1.62 | 0.090 | 0.93 | 2.85 | 0.85 | 0.475 | 0.55 | 1.32 |
| **COPD** | 1.03 | 0.869 | 0.70 | 1.54 | 0.82 | 0.467 | 0.49 | 1.39 | 0.88 | 0.483 | 0.62 | 1.25 |
| **Pulmonary hypertension** | 1.00 | xx | xx | xx | 0.85 | 0.559 | 0.50 | 1.46 | 0.64 | 0.017 | 0.44 | 0.92 |
| **Renal disease, GFR <15mg/dl** | 2.62 | 0.001 | 1.50 | 4.58 | 1.83 | 0.468 | 0.36 | 9.37 | 2.63 | 0.000 | 1.69 | 4.10 |
| **Renal disease, GFR <30mg/dl** | 1.44 | 0.149 | 0.88 | 2.36 | 1.26 | 0.785 | 0.24 | 6.45 | 1.62 | 0.018 | 1.09 | 2.40 |
| **Atrial fibrillation** | 1.38 | 0.016 | 1.06 | 1.79 | 1.74 | 0.001 | 1.26 | 2.40 | 2.03 | 0.000 | 1.61 | 2.57 |
| **Diabetes mellitus** | 0.84 | 0.206 | 0.64 | 1.10 | 0.96 | 0.803 | 0.70 | 1.31 | 1.00 | xx | xx | xx |
| **Emergency** | 1.33 | 0.209 | 0.85 | 2.09 | 1.60 | 0.084 | 0.94 | 2.74 | 1.24 | 0.244 | 0.86 | 1.77 |
| **Number of cases per center** | 1.00 | 0.024 | 1.00 | 1.00 | 1.00 | 0.859 | 1.00 | 1.00 | 1.00 | 0.003 | 1.00 | 1.00 |

xx: The Research Data Center of the Federal Bureau of Statistics censored all values that could allow conclusions to be drawn about a single patient or a specific hospital.

CABG: coronary artery bypass graft; CAD: coronary artery disease; COPD: chronic obstructive pulmonary disease; EuroSCORE: European System for Cardiac Operative Risk Evaluation; GFR: glomerular filtration rate; MI: myocardial infarction;
NYHA: New York Heart Association; SAVR: surgical aortic valve replacement; SD: standard deviation; TAVR: transcatheter aortic valve replacement.
